# Matrix Metalloproteinase Expression in Contusional Traumatic Brain Injury: A Paired Microdialysis Study

**DOI:** 10.1089/neu.2014.3764

**Published:** 2015-10-15

**Authors:** Mathew R. Guilfoyle, Keri L.H. Carpenter, Adel Helmy, John D. Pickard, David K. Menon, Peter J.A. Hutchinson

**Affiliations:** ^1^ Division of Neurosurgery, Department of Clinical Neurosciences, University of Cambridge, Cambridge, United Kingdom.; ^2^ Division of Anesthesia, Department of Medicine, University of Cambridge, Cambridge, United Kingdom.

**Keywords:** adult brain injury, extracellular matrix, microdialysis

## Abstract

Matrix metalloproteinases (MMPs) are extracellular enzymes that have been implicated in the pathophysiology of blood–brain barrier (BBB) breakdown, contusion expansion, and vasogenic edema after traumatic brain injury (TBI). Specifically, in focal injury models, increased MMP-9 expression has been observed in pericontusional brain, and MMP-9 inhibitors reduce brain swelling and final lesion volume. The aim of this study was to examine whether there is a similarly localized increase of MMP concentrations in patients with contusional TBI. Paired microdialysis catheters were inserted into 12 patients with contusional TBI (with or without associated mass lesion) targeting pericontusional and radiologically normal brain defined on admission computed tomography scan. Microdialysate was pooled every 8 h and analyzed for MMP-1, -2, -7, -9, and -10 using a multiplex immunoassay. Concentrations of MMP-1, -2, and -10 were similar at both monitoring sites and did not show discernible temporal trends. Overall, there was a gradual increase in MMP-7 concentrations in both normal and injured brain over the monitoring period, although this was not consistent in every patient. MMP-9 concentrations were elevated in pericontusional, compared to normal, brain, with the maximal difference at the earliest monitoring times (i.e., <24 h postinjury). Repeated-measures analysis of variance showed that MMP-9 concentrations were significantly higher in pericontusional brain (*p*=0.03) and within the first 72 h of injury, compared with later in the monitoring period (*p*=0.04). No significant differences were found for the other MMPs assayed. MMP-9 concentrations are increased in pericontusional brain early post-TBI and may represent a potential therapeutic target to reduce hemorrhagic progression and vasogenic edema.

## Introduction

Focal traumatic brain contusions display a variable degree of progression during the hours and days after the primary injury. Hemorrhagic expansion of the contusion core and elevation of intracranial pressure (ICP) as a result of pericontusional edema are major contributors to secondary brain injury.

Matrix metalloproteinases (MMPs) are a family of over 20 extracellular endopeptidases that cleave a wide range of protein substrates in diverse signaling pathways.^1^ In particular, the subfamily of gelatinases, MMP-2 (gelatinase A) and MMP-9 (gelatinase B), have been implicated as key mediators of proteolytic blood–brain barrier (BBB) disruption associated with traumatic injury,^2^ ischemia,^3^ and neuroinflammatory disorders.^4^

In the rat cortical contusion model, MMP-9 expression was increased in lesioned tissue, compared to contralateral uninjured brain, and was associated with local BBB permeability and edema.^5^ Correspondingly, an MMP inhibitor (GM6001) reduced the degree of BBB leakage and the extent of edema. In a similar mouse model, MMP-9 knockout animals were found to have smaller final lesion volume and better functional recovery, compared to wild-type mice.^2^

Tissue samples from patients requiring surgical resection of brain contusions have been shown to have significantly higher expression of MMP-9, compared with lobectomies performed for nontrauma indications.^6^ Further, *in vivo* microdialysis studies in traumatic brain injury (TBI) patients have also shown increased concentrations of MMP-9 and, possibly, MMP-2 early postinjury.^7,8^ However, it is unclear from human studies to date whether MMP-9 overexpression in TBI patients is localized to pericontusional brain, as found in animal models, or is a more generalized response to trauma.

This study sought to address this question by monitoring the temporal and spatial concentration of selected MMPs (-1, -2, -7, -9, and -10) in TBI patients using paired microdialysis catheters inserted simultaneously within pericontusional and radiologically normal brain.

## Methods

All study protocols were approved by the East of England (Essex) NHS Research Ethics Committee (ref 11/EE/0075). Assent from patients' next of kin was obtained. Eligible patients were adults (>18 years) admitted post-TBI with contusions evident on computed tomography (CT) imaging and requiring neurointensive care treatment. Patients were classified as having severe injury if the presenting Glasgow Coma Scale (GCS) score was less than or equal to 8.^9^ Severity of injury based on the initial computed tomography (CT) scan was graded using the modified Marshall scoring system.^10^ All patients received sedation (with or without neuromuscular blockade) and mechanical ventilation, together with multi-modality monitoring, and were managed according to a standardized tiered therapy protocol.^11^ Recovery at 6 months was measured on the Glasgow Outcome Scale (GOS) and dichotomized as good (GOS 4 or 5) or poor (GOS 1–3) outcome.^12^

### Monitoring

Invasive neuromonitoring was inserted at two sites for each patient with the intention of having a microdialysis catheter within radiologically normal white matter and another within pericontusional brain, but avoiding the hemorrhagic core. On admission, a triple-lumen cranial access device (Technicam, Newton Abbot, UK) was placed in the right or left frontal region as standard in our unit. An ICP monitor (Codman, Raynham, MA), a brain-tissue oxygen probe (Licox Neurosciences, Andover, UK), and a microdialysis catheter (CMA 71; 100-kDA molecular weight cutoff) perfused with 3.5% (w/v) human albumin solution (Pharmacy Manufacturing Unit, Ipswich Hospital NHS Trust, Ipswich, UK) in central nervous system (CNS) perfusion fluid were introduced through the access device. After assent, up to three further invasive monitors, at least one of which was a microdialysis catheter (CMA 71 and perfused with 3.5% albumin solution, as described above) were placed in proximity to a contusion, either through a second cranial access device or twist drill holes. If the first set of monitors happened to be placed adjacent to a contusion, the second set were inserted in radiologically normal brain on the contralateral frontal region. In patients requiring an emergent craniotomy for an acute subdural hematoma (SDH), the pericontusional monitoring was placed adjacent to underlying contusions under direct vision at the end of surgery, tunneled through the scalp.

### Sample analysis

Hourly microdialysates were pooled into 8-h samples. All samples were analyzed using the Milliplex Multi-Analyte Profiling Human MMP five-plex (MMP-1, MMP-2, MMP-7, MMP-9, and MMP-10) analyte premixed kit (Millipore, St Charles, MI), according to the manufacturer's instructions. All samples were assayed in duplicate wells (25 μL per well), and the mean of these results was used. Plates were read using a Luminex 200 analyzer (Luminex Corporation, Austin, TX) running STarStation software (Applied Cytometry Systems, Sheffield, UK). Protein concentrations were calculated by reference to an eight-point spline fit curve for each MMP.

### Statistical analysis

To mitigate the effects of different monitoring periods in each patient, the mean concentrations of each MMP in the first 72 h postinjury (<72 h) and in the subsequent 72 h (>72 h) were calculated for each patient. Repeated-measures analysis of variance (ANOVA) with MMP concentration as the dependent variable, and site of monitoring (normal vs. injured) and time (<72 h vs. >72 h) as the independent variables, was then applied for each MMP separately. Univariate subgroup comparisons were analyzed with the independent-samples *t*-test. All calculations were performed in R software (v3.0.2, www.r-project.org) and considered significant at 5%.

## Results

Twelve patients (10 male; mean age, 46 years; range, 21–65) were enrolled (Table 1). Paired microdialysis monitoring was commenced a mean of 36 h (range, 16–48) postinjury. No complications attributable to the additional study monitoring were observed. Two patients died during their intensive care treatment as a result of refractory intracranial hypertension. Of the 10 patients who survived, 7 had a favorable outcome (GOS 4–5, moderate disability or good recovery) at 6 months follow-up, and 3 were severely disabled (GOS 3)

**Table 1.** Details of the Patient Cohort

*No.*	*Sex*	*Age (years)*	*Mechanism of Injury*	*GCS at presentation*	*Pupil reaction (R/L)*	*CT scan*^a^	*Evacuated SDH*	*GOS*^b^*(6 months)*
1	M	28	Fall	8	+/+	2d	—	4
2	M	67	Fall	6	+/+	5b	Right	3
3	M	31	Fall	5	−/+	3	—	3
4	M	67	Pedestrian RTA	10	+/+	2c	—	5
5	F	59	Fall	12	+/−	2d	—	1 (10 days)
6	M	65	Fall	11	+/+	2d	—	4
7	M	55	RTA	9	+/+	5b	Left	5
8	F	42	Fall	3	−/+	5b	Left	3
9	M	30	RTA	7	+/+	5b	Right	5
10	M	22	Assault	8	+/+	5b	Right	4
11	M	42	Fall	7	+/+	5b	Left	5
12	M	48	Assault	10	+/+	2d	—	1 (7 days)

^a^ Modified Marshall computed tomography classification.^10^

^b^ 5=good recovery, 4=moderate disability, 3=severe disability, 2=vegetative state, 1=death.^12^

GCS, Glasgow Coma Scale^9^; R, right; L, left; CT, computed tomography; SDH, subdural hematoma; GOS, Glasgow Outcome Scale; RTA, road traffic accident.

**FIG. 1.**
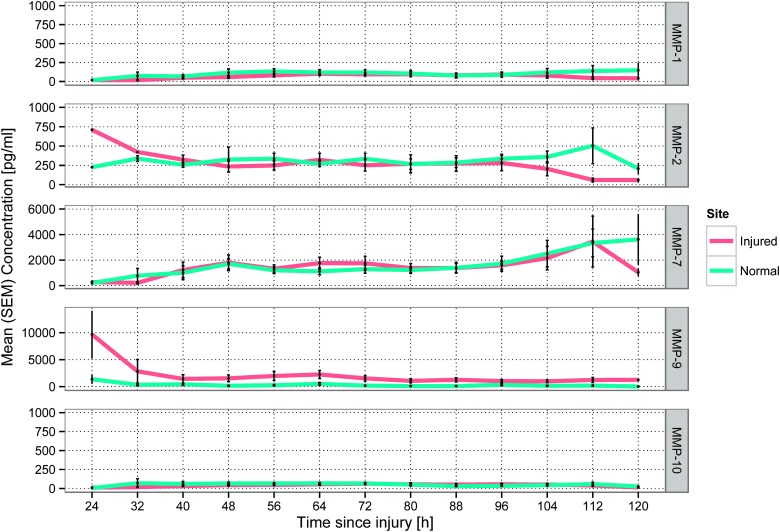
Mean time-concentration curves for each MMP across all patients. Error bars represent standard error of the mean (SEM). MMP, matrix metalloproteinase. Color image is available online at www.liebertpub.com/neu

**FIG. 2.**
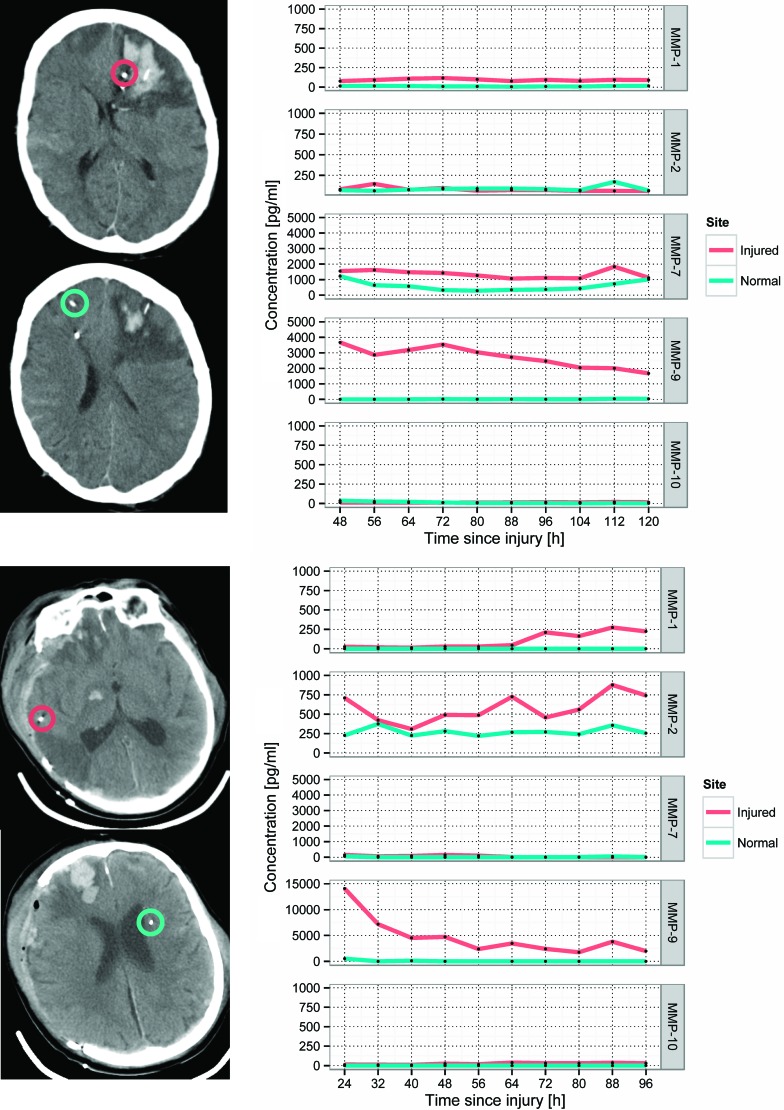
Examples of patient computed tomography scans demonstrating catheter placement and corresponding time-concentration curves for each MMP. Top: patient 5; bottom: patient 2 (see Table 1). Red circles highlight the tip of the microdialysis catheter in pericontusional brain; green circles indicate the microdialysis catheter in normal brain. MMP, matrix metalloproteinase; SEM, standard error of the mean. Color image is available online at www.liebertpub.com/neu

Mean time-concentration plots across for each MMP assayed are shown in Figure 1. Examples of catheter placement and corresponding individual time courses of the five MMPs assayed are shown in Figure 2. Although, in some patients, MMP-2 concentrations gradually increased in pericontusional brain, overall concentrations of MMPs -1, -2, and -10 were similar at both sites during the monitoring period and did not demonstrate consistent temporal trends across the cohort of patients. There was a gradual increase in the concentration of MMP-7 at both monitoring sites, but this was not observed in all patients.

In contrast, MMP-9 concentrations were consistently higher in the injured brain, compared to radiologically normal brain. The highest concentrations of MMP-9 were observed at the earliest monitoring times (i.e., <24 h) within injured brain. Thereafter, MMP-9 concentrations decreased, but remained higher, within injured brain, compared to radiologically normal brain, to 72 h postinjury and beyond.

**FIG. 3.**
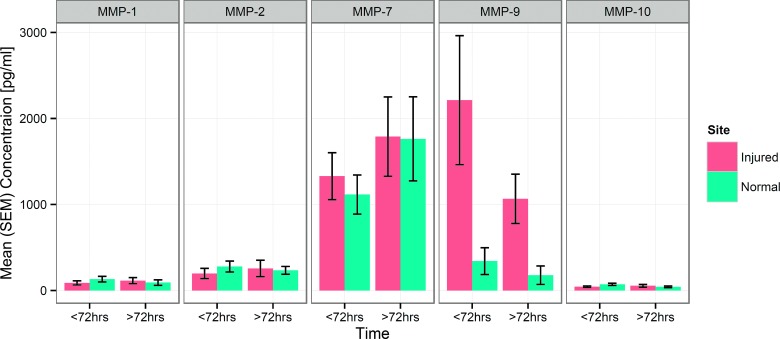
Group mean (SEM) concentration of each MMP at each monitoring site <72 h postinjury and >72 h injury. Repeated-measures analysis of variance demonstrated significant differences between monitoring sites (*p*=0.03) and between monitoring periods (*p*=0.04) for MMP-9; no significant differences were observed for MMP-1, -2, -7, and -10 (see text for full details). MMP, matrix metalloproteinase; SEM, standard error of the mean. Color image is available online at www.liebertpub.com/neu

Mean concentrations of the five MMPs in normal and injured brain in the periods <72 and >72 h postadmission are shown in Figure 3. Repeated-measures ANOVA showed no significant difference in MMP-1, -2, -7, or -10 concentrations between normal or injured sites and no effect of time. However, MMP-9 concentrations were significantly higher in pericontusional brain, compared to radiologically normal brain (*p*=0.03), and were also significantly higher in the early monitoring period (<72 h), compared with later time points (>72 h; *p*=0.04).

There were no significant differences (*p*>0.05) in concentrations of any of the assayed MMPs when the cohort of patients was divided into two groups based either on presenting severity of TBI (GCS ≤8 vs. >8), whether or not the patient had an SDH evacuated, and functional outcome (GOS 1–3 vs. 4–5).

## Discussion

This study has demonstrated that there is a specific, early, and localized increase in MMP-9 concentrations within pericontusional brain post-TBI. Although the concentration of MMP-7 appeared to gradually increase over the monitoring period in both injured and uninjured brain, no significant differences were found in the concentrations of MMP-1, - 2, -7, and -10 with respect to monitoring site or time. Comparing patient groups based on presenting GCS, presence of subdural haematoma requiring evacuation, and functional outcome, found no significant differences in MMP concentrations. However, it is unsurprising that in a relatively small cohort of patients there is no statistical relationship between interstitial MMP concentrations and clinical features that are dependent on a plurality of factors.

Our findings are in accord with pre-clinical evidence that MMP-9 expression is increased in perilesional brain after experimental brain contusion and, in these models, corresponds with the development of BBB permeability and expansion of final lesion volume.^2,5^ Mechanistically, MMPs have been shown to directly disrupt tight junctions between endothelial cells through cleavage of the extracellular domains of critical structural proteins, including claudin and occludin.^13,14^ This process dramatically alters water reflectance and solute permeability of the BBB, exacerbating vasogenic edema, and also contributing to immune cell invasion into the CNS. These molecular events provide a framework for the microvascular failure, which is thought to underlie contusion expansion.^15^ Multiple upstream signaling molecules have been shown to have a role in regulating expression and activation of MMP-9, including cytokines (e.g., interleukin-1β), growth factors (e.g., transforming growth factor TGF-β and vascular endothelial growth factor VEGF), neurotransmitters and small molecules (e.g., histamine and nitric oxide), and hormones (e.g., epinephrine).^16^ In the wider experimental brain injury literature, MMPs have been identified as mediators of lesion expansion and perilesional edema in models of ischemic stroke and intracerebral hemorrhage.^17,18^ Importantly, recent evidence has highlighted the crucial roles that MMPs have in the subacute and chronic reparative processes after brain injury, such as neurovascular remodeling and migration of cells from the subventricular zone to damaged tissue.^19,20^

Grosslete and colleagues and Zheng and colleagues have previously reported elevated levels of MMP-9 in cerebrospinal fluid (CSF) sampled acutely from TBI patients by ventriculostomy, with comparable temporal profiles demonstrating greatest concentrations in the first sample at 24 h postinjury followed by a decline, but remaining higher than control CSF for at least 72 h.^21,22^ Vilalta and colleagues studied 4 patients with diffuse brain injury using microdialysis and found similar early elevation of cerebral MMP-9 concentrations.^7^ More recently, Roberts and colleagues conducted a single-site microdialysis study of MMP-1, -2, -3, -7, -8, and -9 in 8 TBI patients with a mixture of diffuse and focal injuries; similarly to the present study, they found that MMP-9 was increased early postinjury and thereafter declined.^8^

The present data both corroborate these temporal MMP-9 profiles in a larger cohort of TBI patients and add the important new finding that, in patients with predominantly focal or contusional injury, MMP-9 response is localized to perilesional brain. The paired catheter design means that it is far less likely that the observed changes in MMP-9 are attributable to artefact from catheter insertion, given that a similar effect would have been observed at both monitoring sites. The evidence from this study goes some way to supporting the hypothesis that the mechanisms of MMP-9-induced BBB disruption and edema elucidated in animal models are also relevant in human TBI. Indeed, the temporal pattern of MMP-9 changes is consistent with MRI studies of contusion expansion in humans.^23^

Both previous microdialysis studies have found evidence for increased cerebral expression of MMP-2 post-TBI, although Vialta and colleagues identified an early peak similar to MMP-9, whereas in the later study by Roberts and colleagues, MMP-2 concentration was initially low and increased at approximately 48 h postinjury, with a subsequent decrease.^7,8^ In the present study, patients exhibited variable MMP-2 responses, with no consistent spatiotemporal pattern emerging on averaging across the cohort. Interestingly, an earlier study of contusion resection tissue also found MMP-2 concentrations that varied widely, and the resulting overall difference in expression, compared to control patient samples, was modest.^6^ Together, these findings suggest that MMP-2 concentrations may be significantly elevated in only a subset of TBI patients; whether this is a function of injury severity or other patient-specific clinical factors will require larger studies to resolve. The relative importance of MMP-2 compared with MMP-9 in the pathogenesis of contusion expansion and brain edema is unclear, and there is conflicting evidence on the role of MMP-2 in exacerbating lesion volume from different brain injury models.^2,24^ However, because both MMP-2 and -9 are gelatinases and act on similar substrates, they may represent redundant pathways in post-traumatic proteolytic breakdown of the BBB. If interventions targeted at MMP activity are to be successful, a more complete understanding of the role of MMP-2 will be essential, particularly given that evidence from pre-clinical TBI models suggests that MMP-2 and MMP-9 show different responses to current therapies, such as hypothermia.^25^

This study has a number of limitations. First, no patient was monitored within the first 12 h of injury, and therefore the expression pattern of MMPs in the very acute stage remains uncertain. Second, MMP activity is regulated by endogenous tissue inhibitor of metalloproteinase (TIMP) proteins, which bind with latent and activated MMPs.^1^ Although MMP-TIMP complexes would likely exceed the 100-kDa cutoff of the CMA 71 microdialysis catheter, it cannot be determined from the present data whether all MMP-9 assayed was enzymatically active; future studies to address this question will be challenging owing to low absolute amount of protein recovered with microdialysis. Third, the findings of the current study injury do not speak to whether MMP concentrations will display similar temporal profiles in patients with a predominantly diffuse axonal injury pattern on initial CT.

Induced hypothermia has been shown to attenuate MMP-9 expression in rodent TBI and stroke models, but, as yet, this phenomenon has not been investigated in human patients.^25,26^ Despite a number of randomized trials, therapeutic hypothermia has not been conclusively shown to improve outcome post-TBI, though its use to control ICP remains widespread.^11,27,28^ Conceivably, targeting hypothermia to patients with increased MMP-9 expression may be a method to select the subgroup of patients in which the benefits of this treatment outweigh its deleterious effects.

MMP inhibitors have been investigated as promising therapies for a number of diseases, most notably in metastatic cancer. However, despite successful pre-clinical findings, early-phase clinical trials in cancer patients have proven disappointing owing to lack of efficacy and excessive adverse effects.^29^ Of interest, tetracycline antibiotics, most notably doxycycline, inhibit MMP-2 and -9 through chelation of the zinc ion in the catalytic site.^30^ Doxycycline, at subantimicrobial doses, has been shown to be of benefit in periodontitis, likely through its effect on MMP-9 activity.^31^ On the same basis, doxycycline has also been suggested as a treatment to stabilize growth of abdominal aortic aneurysms, although the evidence to date is conflicting.^32^ Currently available MMP inhibitors are not selective for specific subtypes, but, at the same time, are not equally active on all members of the enzyme family; combined with redundancy in MMP pathways, this may be an explanation for the limited efficacy of MMP inhibitors in clinical studies to date.

This study suggests that MMP-9 may be a therapeutic target to reduce lesion progression and brain swelling in contusional TBI. Investigating the efficacy of MMP inhibitors in this context will first require further detailed studies of the association between pericontusional MMP-9 concentration, contusion expansion and vasogenic edema, ICP and treatment intensity, and cerebral metabolism to establish robust measures of efficacy. Human studies to date indicate that the useful therapeutic window for MMP-9 inhibition post-TBI may extend up to 72 h postinjury, but it is likely that any agent will be most effective at minimizing brain edema and contusion expansion if administered as acutely as possible. Defining the appropriate duration of MMP inhibition will also need to consider the later role these enzymes have in postinjury CNS repair.
